# Pathological and Physiological Significance of Hippocampal Ripples in Epilepsy

**DOI:** 10.31662/jmaj.2026-0131

**Published:** 2026-06-12

**Authors:** Takamitsu Iwata

**Affiliations:** 1 Department of Neurosurgery, Graduate School of Medicine, The University of Osaka, Suita, Osaka, Japan; 2 Department of Neurosurgery, Sakai City Medical Center, Sakai, Osaka, Japan

**Keywords:** high-frequency oscillations, sharp-wave ripple, hippocampus, epilepsy, memory, intracranial electroencephalography (EEG)

## Abstract

Ripple activity is defined as a high-frequency synchronous neural activity that has emerged as an important biomarker of epilepsy, both in research and clinical practice. In intracranial electroencephalography, ripple-band activity is closely linked to epileptogenicity and has been investigated as a marker to guide localization of epileptogenic tissue and predict surgical outcomes. Simultaneously, in the hippocampus, ripple activity additionally includes physiological sharp-wave ripples (SWRs), which play essential roles in memory consolidation, retrieval, and the selection of experiences for long-term storage. This overlap gives hippocampal ripple activity dual clinical significance: it may indicate pathological synchrony related to epilepsy, and it can also indicate the physiological processes that support memory and other higher cognitive functions. This review summarizes the current knowledge regarding the dual significance of hippocampal ripple activity in epilepsy. First, the pathological relevance of high-frequency oscillations as biomarkers of epileptogenicity and the physiological role of SWRs in hippocampal-cortical information processing are outlined. Subsequently, the methodological challenges involved in distinguishing pathological ripples from physiological SWRs in human intracranial recordings are discussed. Recent advances in consensus-based criteria, spatiotemporal analyses, and machine learning–based detection methods are further reviewed. Mechanistic frameworks further indicate that ripples reflect a shared synchrony-generating substrate, whose meaning depends on the network state, behavioral context, and spatial relation to the epileptogenic focus. Finally, ripple activity could be interpreted not only as a local hippocampal event but also as a marker of hippocampo–neocortical network state, with potential implications for epileptogenicity assessment, surgical planning, and function-preserving treatment strategies. Understanding the dual role of the hippocampal ripple activity may provide a basis for more precise epilepsy therapies that balance seizure control and memory preservation.

## Background

Surgical treatment and neurostimulation are important therapeutic options for drug-resistant focal epilepsy and can improve not only seizure frequency but also quality of life. In temporal lobe epilepsy, memory impairment is likely to co-occur with hippocampal lesions (e.g., hippocampal sclerosis or tumor) and network dysfunction, while therapeutic interventions can potentially alter memory function. A fundamental challenge in epilepsy treatment is simultaneously achieving seizure control and preserving brain function. This is particularly important in temporal lobe epilepsy, where treatment strategies are required to sufficiently control seizure onset and the seizure network while minimizing the impairment of higher-order functions, including memory ^(1), (2)^. A key issue in achieving this is how to interpret hippocampal ripple activity, a form of fast synchronous neural activity closely linked to both epileptogenicity and memory function. Ripples are classified as pathological high-frequency oscillations (HFOs) that reflect epileptogenicity, but they also occur as physiological sharp-wave ripples (SWRs) that are important for memory consolidation. Taken together, current results indicate that suppressing ripples may suppress epileptic seizures but at the same time carries the risk of impairing memory function. This duality is a major challenge in epilepsy treatment and has attracted both academic and clinical interest ^(3)^. The concept of a more selective suppression of pathological HFOs while protecting SWRs has been emphasized in recent years as key to minimizing this trade-off ^(4)^.

In hippocampal epilepsy, ripples are state-dependent biomarkers, the meaning of which depends on whether they reflect physiological memory-related synchrony or pathological epileptogenic synchrony. In this article, we review how ripple activity in hippocampal epilepsy may be interpreted as a biomarker of the network state and discuss its implications for epileptogenicity, memory function, and treatment.

## Foundational Concepts: Pathological HFOs and Physiological SWRs

### Pathological HFOs: Positioning as a biomarker of epileptogenicity

In general, HFOs refer to activity in the 80–500 Hz band. In clinical contexts, they are often classified as ripples (80–250 Hz) and fast ripples (250–500 Hz). Fast ripples, which are more common, are considered to have stronger pathological significance. However, because physiological SWRs in the hippocampus also fall within the ripple band, it is difficult to distinguish between pathological and physiological activity based solely on the amplitude or frequency band ^(3), (5)^. HFOs are observed not only in the hippocampus but also in the neocortex and deep nuclei. Mechanistically, in epileptic networks, a breakdown of inhibitory control and alterations in synchronizing mechanisms have been suggested to shift normal ripple-like activity toward more pathological HFOs, such as fast ripples ^(6), (7)^.

A substantial body of research has indicated that HFOs observed near the epileptic focus are candidate markers of epileptogenicity, while a close relationship has been reported between resection of HFO-generating regions and outcomes of epilepsy surgery ^(8), (9)^. Meta-analyses have further shown that postoperative outcomes are favorable in cases in which HFO-generating regions can be sufficiently resected, thus supporting the idea that HFOs contain information about regions that should be removed ^(10)^.

### Hippocampal SWRs: A core phenomenon linked to memory consolidation and retrieval

Understanding of SWRs was advanced by the discovery that hippocampal place cells encode specific locations and that their firing sequences are replayed during rest and sleep. SWRs are synchronous high-frequency events observed in the CA1 region involved in the selection and consolidation of experiences ^(11)^. Animal studies have shown that the selective suppression of SWRs impairs postlearning memory consolidation, with reduced performance when SWRs are suppressed during sleep/rest; conversely, the prolongation of SWRs improves accuracy in memory tasks, supporting the causal role of SWRs in memory consolidation ^(12), (13), (14)^. In particular, the hierarchical coupling of slow oscillations, spindles, and hippocampal ripples during sleep has been observed in humans, supporting models of hippocampal-neocortical information transfer and memory consolidation ^(15), (16)^.

**Figure 1.**
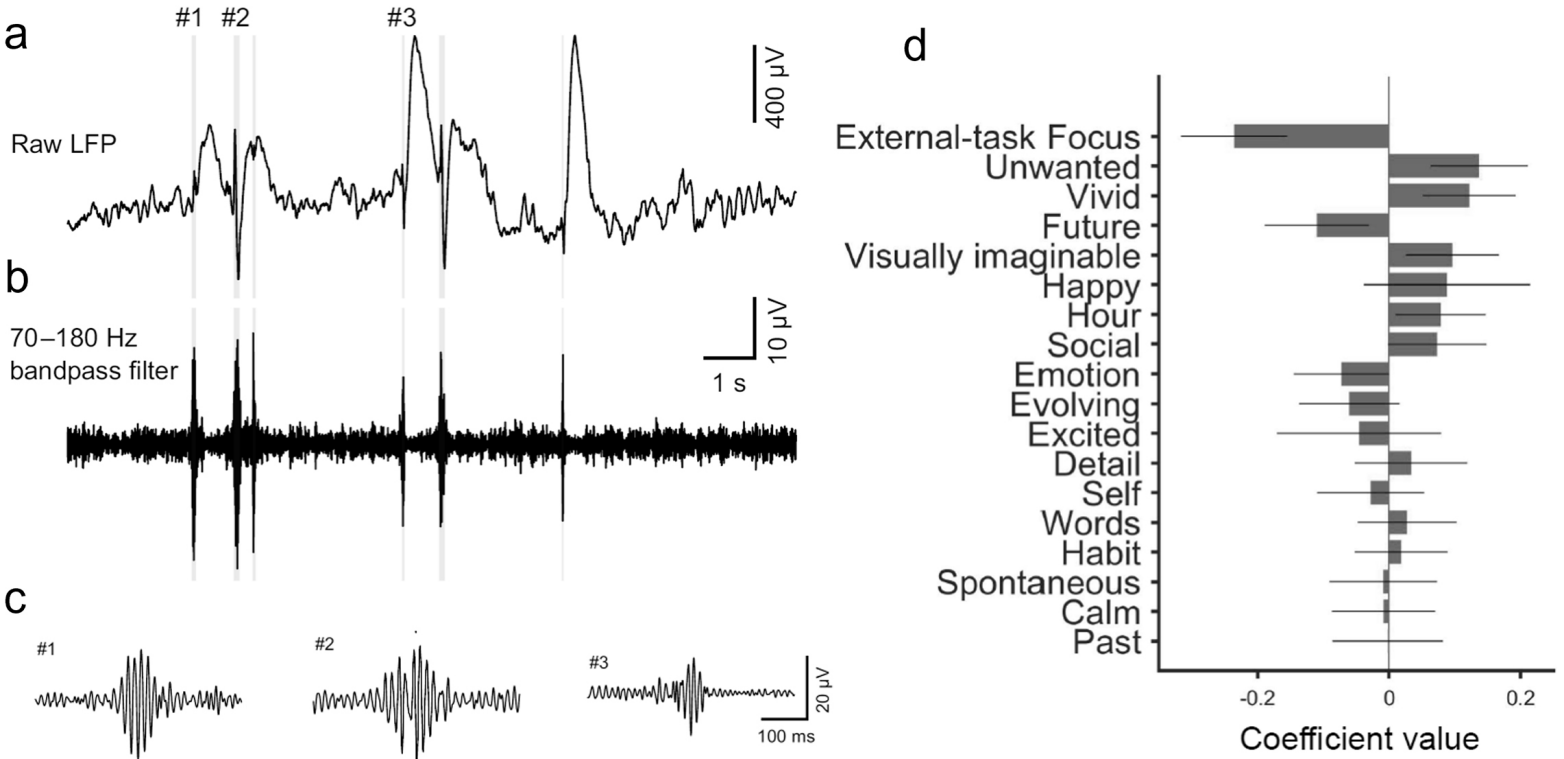
Detection of hippocampal SWRs and their association with thought content. (a) Unfiltered hippocampal LFP; detected SWR events are indicated by shaded regions. (b) The bandpass filtered signal in the ripple band (70–180 Hz). (c) Representative waveforms of detected ripple events. (d) Results of a mixed-effects general regression analysis examining the association between SWR rate and thought/emotional states assessed by simultaneous experience sampling; regression coefficients are shown (adapted from Iwata et al. ^(19)^; licensed under a Creative Commons Attribution 4.0 International License [CC BY 4.0]. The original figure has been partially modified and converted into grayscale). LFP: local field potential; SWR: sharp-wave ripple.

In humans, hippocampal SWRs are associated with the encoding and retrieval of episodic memory, while several reports have indicated that SWR frequency and coupling with the cortex predict subsequent successful recall ^(17), (18)^. SWRs occur not only during sleep but also during awake rest and are thought to support future planning, decision-making, and self-generated thought through the reactivation of past experiences ^(19), (20), (21)^. More recently, studies have indicated that awake SWRs are involved in the selection of experiences retained as memories, thereby updating the view of SWRs as merely an offline phenomenon ^(22)^. Investigating these roles in humans has historically been challenging because long-term, high–temporal-resolution recordings of hippocampal activity are limited by invasiveness, recording duration, noise, artifacts, and the coexistence of pathological high-frequency discharges. Nevertheless, a recent study has achieved stable long-term detection and quantification of human hippocampal SWRs and has combined these recordings with experience-sampling approaches to quantify ongoing thought content. This approach demonstrates that SWRs occur preferentially during specific internally generated thought states, thus extending the conventional view that links SWRs to memory consolidation (Figure 1). These findings indicate that hippocampal SWRs provide a window into higher-order cognitive dynamics in humans, thereby enabling the empirical investigation of internal thought processes through deep neural activity.

Thus, in the hippocampus, pathological ripples and physiological SWRs may overlap in morphology and context; however, because their clinical significance is opposing, standardization of detection and discrimination is essential.

## Standardization of Measurement, Detection, and Definitions

Without reliable discrimination, ripple-based interpretation risks overestimating epileptogenic tissue and underestimating physiologically relevant hippocampal activity. In clinical intracranial electroencephalography (EEG), many factors can distort detection, including references, filters, artifacts (electromyography and device noise), and epileptic discharges ^(23)^. To date, methods have been proposed to discriminate SWRs from noise and pathological events by combining time–frequency analysis, phase–amplitude coupling, event morphology (sharp-wave co-occurrence, slow wave–spindle synchrony, multicyclicity, and narrowband frequency peaks), localization (e.g., confinement to the CA1 pyramidal cell layer), co-occurrence (spike-on-ripple and synchrony with interictal epileptiform discharges), and behavioral state (sleep stage, wakefulness, and memory); however, standardization robust enough for clinical implementation has not yet been achieved ^(3), (24)^.

In recent years, machine learning–based analytical platforms that can take the diversity of SWR waveforms into account have been increasingly developed, and efforts to establish reproducible and consistent detection methods are advancing ^(25)^. In addition, a consensus has been reached that summarizes the practical recommendations for distinguishing hippocampal SWR detection from other high-frequency activities ^(3)^.

More recently, methodological caution has been raised indicating that the detection of human ripple-like activity may be influenced by background activity and 1/f components, and careful interpretation and comparison are required ^(26)^. As such, detection and classification are limited when relying only on threshold-based bandpass plus envelope methods, while automated detection using machine learning has advanced.

## Mechanisms: Why Do Ripples Appear in Both Pathology and Physiology?

The hippocampus is the core of a broad network spanning the medial temporal lobe and cortex that supports episodic memory. In mesial temporal lobe epilepsy, this network often becomes epileptogenic and is reorganized into pathological connectivity such that the hippocampus commonly behaves as a network node ^(27)^. SWRs are highly synchronous events that support hippocampal-cortical information reactivation and contribute to memory consolidation and retrieval. However, in epilepsy, heightened local circuit excitability, reflecting shifts in the excitation–inhibition balance and impaired inhibitory interneuron function, may cause the same synchrony-generating substrate to be biased toward pathological synchrony, thereby promoting epileptogenicity ^(28), (29)^. As such, ripples can be viewed as manifestations of transient synchrony, whose meaning hinges on whether synchrony serves as information reactivation or pathological firing in a manner dependent on the state, context, and spatial proximity to the epileptogenic focus ^(11)^.

Long-term disease duration in mesial temporal lobe epilepsy has been reported to be associated not only with seizures but also with long-term memory impairment, including remote and autobiographical memories ^(30)^. It is plausible that the disruption of SWR-generating mechanisms or a shift toward pathological ripples contributes to these chronic changes; however, further research is required to establish causality.

## Treatment Outcomes of Focal Resection Surgery in the Hippocampus

In mesial temporal lobe epilepsy, particularly cases of hippocampal sclerosis, high levels of seizure suppression can be achieved by resecting the pathological hippocampus ^(31)^. Some reports have indicated that memory function may improve with decreased seizure frequency and reduced interictal activity, indicating an association between reduced network noise and resection ^(32), (33)^. The degree of improvement varies widely across individuals and depends on multiple factors, including laterality, residual hippocampal function, and network reorganization. However, hippocampal resection carries a risk of memory impairment, making preoperative evaluation and risk stratification indispensable. Declines in verbal memory following left temporal lobe resection and the course of long-term network plasticity have been reported ^(34), (35)^, making it necessary to set composite therapeutic goals that do not consider seizure freedom alone a success.

## Coexistence of Pathological Ripples and SWRs

**Figure 2.**
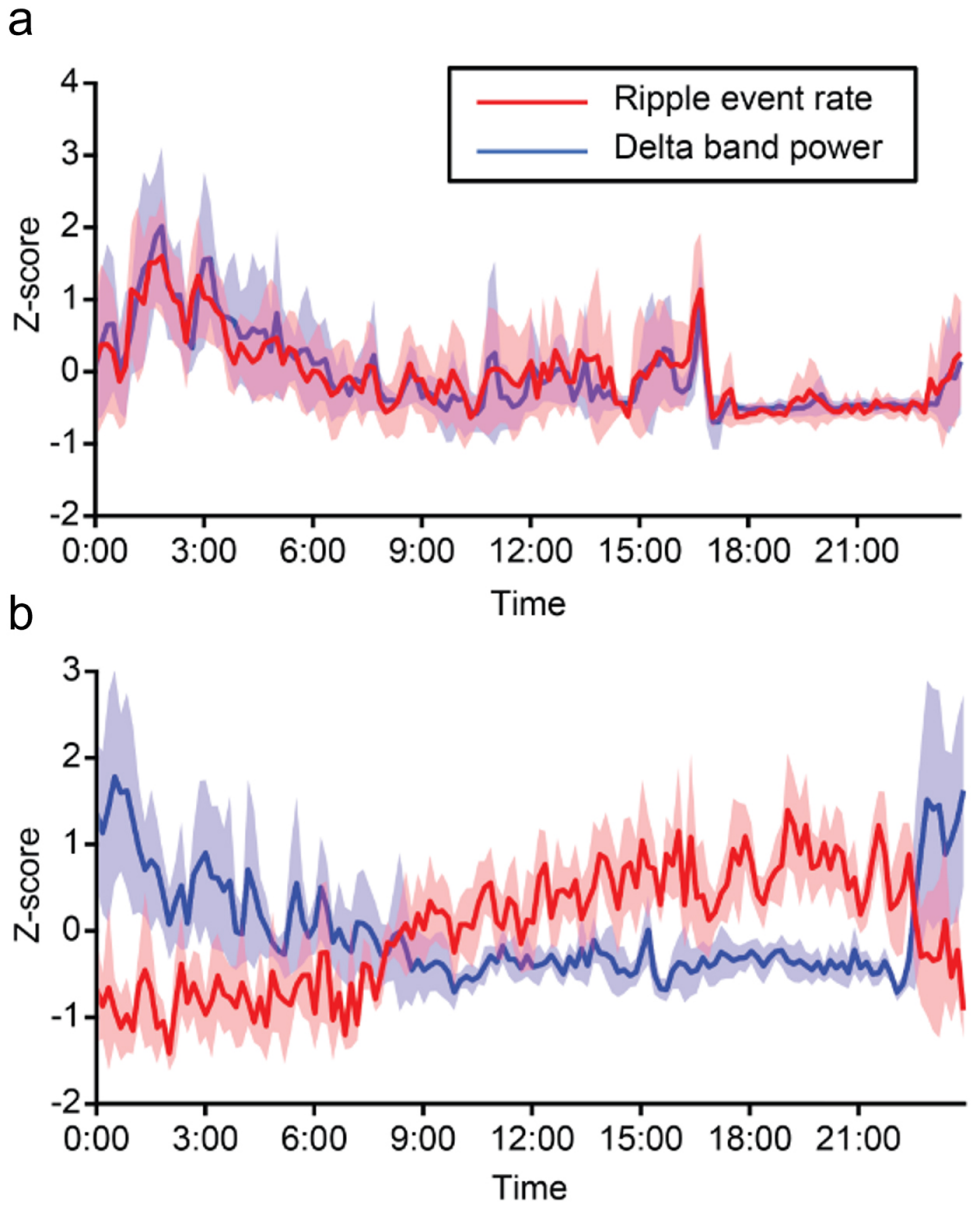
Circadian dynamics of hippocampal ripple activity and cortical delta band power according to the hippocampal epileptogenicity. Lines indicate the mean and 95% confidence intervals of the z-scored hippocampal ripple event rate, including both SWRs and pathological ripples (red), together with cortical delta band power (blue), over a 24-hour period, shown separately for patients with low (a) and high (b) hippocampal epileptogenicity (adapted from “Iwata et al. ^(4)^; licensed under a Creative Commons Attribution 4.0 International License [CC BY 4.0]. The original figure has been partially modified). SWR: sharp-wave ripple.

It is important to understand how the degree of the coexistence of pathological ripples and SWRs reflects the epileptogenic state of the hippocampus. One key conceptual advance has been to move beyond treating hippocampal ripples as purely local events and instead interpreting them in the context of hippocampo–neocortical network dynamics. One reported approach takes advantage of the tendency for SWRs to occur during sleep and rest and to synchronize with low-frequency cortical activity to evaluate hippocampal state based on the correlation between overall hippocampal ripple occurrence and cortical delta band power ^(4)^. By quantifying cortical–hippocampal synchrony as an index of hippocampal–cortical coupling, this framework revealed abnormal synchronization in hippocampal epilepsy, suggesting its potential clinical applicability for assessing hippocampal epileptogenicity (Figure 2). Using this biomarker, hippocampal sclerosis could be detected with high accuracy, while an increase in the seizure burden was associated with a reduction in ripple–delta correlation ^(4)^. These findings indicate that this measure may serve as an indirect indicator of the balance between the physiological SWRs and pathological epileptic ripples in the hippocampus.

## Conclusions

Hippocampal ripple activity has dual significance in temporal lobe epilepsy, reflecting both epileptogenic pathology and the physiological mechanisms of memory. This highlights the need for therapeutic strategies that do not simply suppress ripple activity globally but instead distinguish and preferentially target pathological ripples while preserving physiological SWRs. Advances in standardized detection, classification, and network-level biomarkers may enable the development of ripple-guided therapies. Ultimately, the goal of epilepsy treatment should extend beyond seizure freedom to encompass the preservation of memory and other higher cognitive functions.

## Article Information

### Acknowledgments

The author would like to thank the following principal investigators for their invaluable contributions to this research: Prof. Takufumi Yanagisawa, Prof. Yuji Ikegaya, Prof. Jonathan Smallwood, and Prof. Haruhiko Kishima.

### Author Contributions

Takamitsu Iwata: Conceptualization, literature review, writing – original draft, visualization, and funding acquisition

### Conflicts of Interest

None

### Article Information

This article is based on the study that received the Medical Research Encouragement Prize of the Japan Medical Association in 2025.
